# Flagellar membrane fusion and protein exchange in trypanosomes; a new form of cell-cell communication?

**DOI:** 10.12688/f1000research.8249.1

**Published:** 2016-04-14

**Authors:** Simon Imhof, Cristina Fragoso, Andrew Hemphill, Conrad von Schubert, Dong Li, Wesley Legant, Eric Betzig, Isabel Roditi

**Affiliations:** 1 Institute of Cell Biology, University of Bern, Bern, Switzerland; 2 Graduate School of Cellular and Biomedical Sciences, University of Bern, Bern, Switzerland; 3 Institute of Parasitology, Vetsuisse Faculty, University of Bern, Bern, Switzerland; 4 Division of Molecular Pathobiology, DCR-VPH, Vetsuisse Faculty, University of Bern, Bern, Switzerland; 5 Janelia Research Campus, Howard Hughes Medical Institute, Ashburn, VA, USA

**Keywords:** protein transfer, membrane fusion, cell-cell communication, flagellum, cilium, Trypanosoma, lattice light sheet microscopy, structured illumination microscopy

## Abstract

Diverse structures facilitate direct exchange of proteins between cells, including plasmadesmata in plants and tunnelling nanotubes in bacteria and higher eukaryotes.  Here we describe a new mechanism of protein transfer, flagellar membrane fusion, in the unicellular parasite *Trypanosoma brucei*. When fluorescently tagged trypanosomes were co-cultured, a small proportion of double-positive cells were observed. The formation of double-positive cells was dependent on the presence of extracellular calcium and was enhanced by placing cells in medium supplemented with fresh bovine serum. Time-lapse microscopy revealed that double-positive cells arose by bidirectional protein exchange in the absence of nuclear transfer.  Furthermore, super-resolution microscopy showed that this process occurred in ≤1 minute, the limit of temporal resolution in these experiments. Both cytoplasmic and membrane proteins could be transferred provided they gained access to the flagellum. Intriguingly, a component of the RNAi machinery (Argonaute) was able to move between cells, raising the possibility that small interfering RNAs are transported as cargo. Transmission electron microscopy showed that shared flagella contained two axonemes and two paraflagellar rods bounded by a single membrane. In some cases flagellar fusion was partial and interactions between cells were transient. In other cases fusion occurred along the entire length of the flagellum, was stable for several hours and might be irreversible. Fusion did not appear to be deleterious for cell function: paired cells were motile and could give rise to progeny while fused. The motile flagella of unicellular organisms are related to the sensory cilia of higher eukaryotes, raising the possibility that protein transfer between cells via cilia or flagella occurs more widely in nature.

## Introduction

Intercellular bridges enabling the direct exchange of macromolecules between cells have been described in a diverse set of multicellular and unicellular organisms. These include plasmodesmata in plants, septal pores in fungi and gap junctions and tunnelling nanotubes in animal cells ^1– 5^. Plasmodesmata permit the transfer of transcription factors and mRNAs, triggering developmental programs in neighbouring cells ^6^. In contrast, only small molecules up to 1 kDa can pass through gap junctions. Tunnelling nanotubes (TNT) are dynamic, ultrathin membranous structures that have been observed to form *de novo* when mammalian cells were mixed in culture ^5^. They have been implicated in tissue repair, development and electrical coupling of cells and permit the transfer of whole organelles such as lysosomes or mitochondria, over distances up to several cell diameters ^7– 11^.

Recently it was shown that green fluorescent protein (GFP) can be transferred from cancer cells to epithelial cells and it was postulated that this happens through a transient membrane fusion between the cells ^12^. Intercellular bridges can also be hijacked by pathogens to infect new host cells. TNTs can be involved in the spread of HIV and prions, and plasmodesmata are used by several viruses to spread through the host plant ^13– 15^. Prokaryotes are also capable of direct exchange of macromolecules via intercellular bridges. *Bacillus subtilis* has been reported to exchange proteins and non-conjugative plasmids through TNT-like structures ^16^. In addition, the social bacterium *Myxococcus xanthus* can exchange outer membrane proteins by transient outer membrane fusion ^17, 18^. In summary, targeted exchange of macromolecules by direct cell-cell contact seems to be a widespread in nature. To date, however, no intercellular bridges have been described in protozoa.

*Trypanosoma brucei* is a unicellular eukaryote that causes human sleeping sickness and nagana in domestic animals. The parasite depends on tsetse flies for its transmission. Tsetse flies feed exclusively on mammalian blood and, in the process, can acquire parasites from infected hosts and transmit their progeny to new hosts. In the course of transmission, trypanosomes progress through several distinct life-cycle stages in the bloodstream of their mammalian host and in the alimentary tract of the fly (reviewed in 19). All life-cycle stages are extracellular and all are equipped with a single flagellum containing a canonical 9+2 axoneme and an extra-axonemal structure called the paraflagellar rod ^20^. In addition to its function in motility, the trypanosome flagellum appears to serve as a sensory organelle ^21– 23^.

Trypanosomes can interact with each other as well as with their hosts. In the mammalian bloodstream they extrude extracellular vesicles originating from the flagellar membrane; these can transfer virulence factors from one trypanosome strain to the other and contribute to trypanosome pathogenesis ^24^. Bloodstream form trypanosomes also communicate with each other by a quorum-sensing mechanism that favours chronic infection and host survival ^25, 26^. Proliferative slender bloodstream forms release a soluble factor that promotes their differentiation to non-proliferative stumpy forms. The chemical identity of this factor is unknown, but it can be mimicked by cell-permeable cyclic AMP or AMP analogues ^25, 27^. Stumpy forms are pre-adapted to survive transmission to the tsetse fly and to differentiate to the next stage of the life cycle, the procyclic form, in the insect midgut ^28, 29^. Several years ago it was shown that procyclic trypanosomes exhibit social motility when cultured on a semi-solid surface, in a manner reminiscent of social swarming by bacteria ^30^. This unexpected behaviour shows that procyclic trypanosomes also have the ability to communicate with each other, but the basis of this is largely unknown ^23^. In order to complete transmission via the tsetse, parasites must migrate from the midgut to the salivary glands. This constitutes a population bottleneck and only very small numbers of trypanosomes make this transition ^31^. Once in the glands the parasites attach to the salivary gland epithelium and proliferate as epimastigote forms ^32^. Attachment is mediated by extensive outgrowths of the trypanosome flagellar membrane, which interdigitates between outgrowths of host epithelial cell membranes. The life cycle is completed by an asymmetric division in which one of the progeny is a metacyclic form that can be transmitted to a new mammalian host ^33^.

*Trypanosoma brucei* can undergo genetic exchange in the tsetse fly as a non-essential part of its life cycle ^34, 35^. Both interclonal and intraclonal mating have been reported ^34, 36^. Meiotic markers are expressed by trypanosomes in the salivary glands ^37^ and flies co-infected with trypanosomes expressing either red or green fluorescent proteins can give rise to double-positive “yellow” cells in this compartment ^35^. The current model of mating is that cells in the salivary glands undergo meiosis and produce haploid gametes that first interact via their flagella, then fuse together completely ^38^, but the actual fusion event has not been visualised so far. We report here that procyclic form trypanosomes are able to fuse their flagellar membranes, resulting in the exchange of flagellar and cytoplasmic proteins. No transfer of nuclei or DNA was observed. Flagellar membrane fusion is a transient event and the cells lose the transferred fluorescent protein over time. We postulate that the direct protein transfer reported here is a new form of cell-cell communication and that the detection of double-positive trypanosomes in the fly may not always be related to genetic exchange. Furthermore, the relatedness of the trypanosome flagellum to cilia of higher eukaryotes raises the possibility that intercellular protein transfer by this mechanism might be more widespread in eukaryotic organisms.

## Results

### Yellow trypanosomes are observed in culture

**Figure 1.**
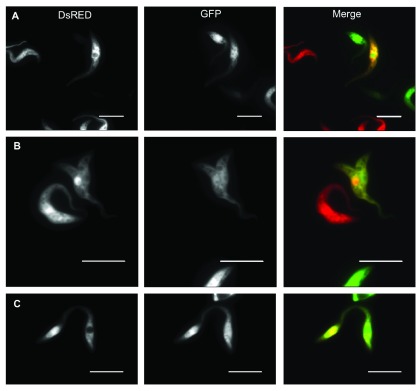
Fluorescence microscopy of co-cultured trypanosomes expressing DsRED or GFP. **A**: Double-positive trypanosome after 24 hours co-culture (WT). **B**: Interacting double-positive trypanosome pair found after 24 hours co-culture (Δproc). **C**: Double-positive trypanosomes connected at their anterior ends (Δproc). The scale bar indicates 10μm. DsRed tends to accumulate in the nuclei of cells that synthesise it, probably because of its propensity to form tetramers.

We initially tagged trypanosomes with different colours in order to study genetic exchange in tsetse flies. For this purpose plasmids encoding different fluorescent proteins (GFP and DsRED) were integrated into defined loci on chromosomes 6 and 10 (see Materials and methods). When flies were co-infected with these tagged procyclic forms, we observed that the growth rates of individual clones differed and one clone/colour overgrew the other. To identify pairs with similar growth rates in culture, pairs of red and green procyclic forms were mixed and their relative numbers monitored by fluorescence microscopy. Unexpectedly, we observed that approximately 1% of the cells were positive for both fluorescent proteins and appeared yellow in merged images. After repeating this experiment several times we discovered that the transfer of cells to fresh medium with fresh FBS led to robust and reproducible production of yellow cells. While most yellow cells observed after 24 hours were single cells with no interacting partner, some were in intimate contact, forming characteristic pairs ( Figures 1A & B). This is in contrast to dividing cells, which have two clearly separate flagella and joined posterior ends ^20^. Yellow cells apparently connected by their anterior ends could also be observed ( Figure 1C).

Procyclic form trypanosomes are covered by several million copies of glycophosphatidylinositol (GPI)-anchored surface glycoproteins known as EP and GPEET procyclins ^39^. To see if these proteins were required for the formation of yellow cells we used a procyclin null mutant (Δproc; ^40^) tagged with either GFP or DsRED. Mixed cultures of the mutant gave rise to double-positive cells at even higher frequencies than wild-type trypanosomes, reaching 5–7% of the population after 24 hours ( Figure S1). No morphological differences could be observed between the procyclin knockout and wild-type parasites or the pairs that they formed. Because of the increased frequency of double-positive cells we used this mutant for some of the experiments reported here. Addition of the chelating agents EDTA or EGTA abolished the formation of yellow cells, indicating a requirement for extracellular calcium ( Figure S1).

### Yellow trypanosomes in culture are not genetic hybrids

Since yellow cells are used as a read-out for mating ^36, 41^, we initially thought that trypanosomes had undergone some form of genetic exchange, although this has never been documented for procyclic forms. The parasites were tagged with different selectable markers (rendering them resistant to blasticidin and G418, respectively), either at the same locus on the diploid copies of a chromosome or at different chromosomal loci. However, despite repeated attempts, we were never able to isolate genetic hybrids that were resistant to both drugs.

**Figure 2.**
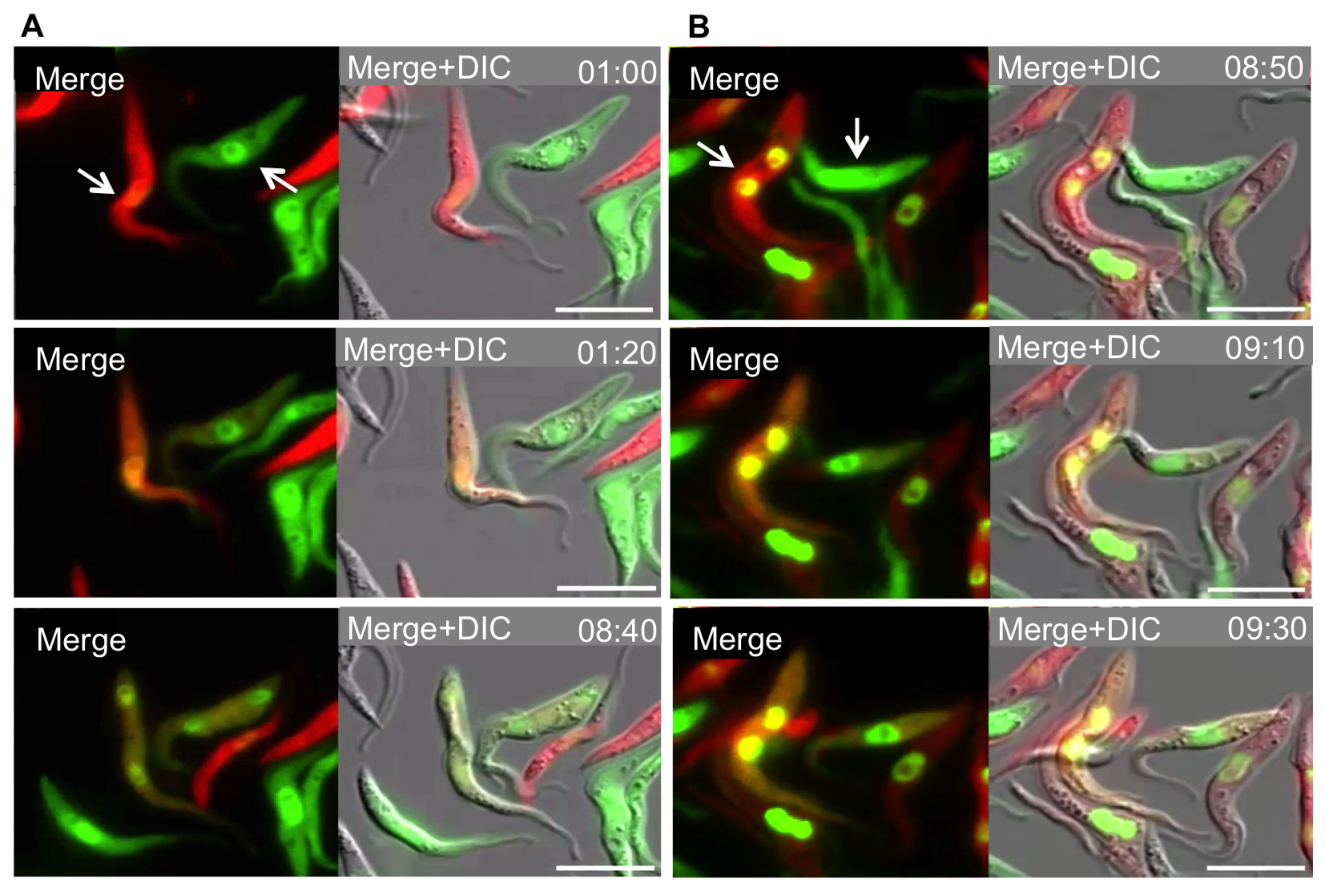
Trypanosomes exchange proteins but not DNA in a contact-dependent manner. Still images from time-lapse fluorescent microscopy of a mixed culture of wild type trypanosomes expressing Histone2B-GFP/cytosolic DsRED or Histone2B-GFP/cytosolic GFP. The time interval between images is indicated at the right corner of the image; the scale bar indicates 10μm; arrows indicate interacting cells. **A**: First image taken after one hour, the cells are clearly separate and only positive for one fluorescent protein in the cytoplasm. Second image 20 minutes later (1:20), cells are in contact and have become positive for DsRED and GFP. Third image, 7 hours and 20 minutes later (8:40), the cells have separated again. ( Video 1) **B**: First image taken after 8 hours 50 minutes, cells are only positive for one fluorescent protein in the cytoplasm. Second image, cells have become double-positive (09:10). Third image, 20 minutes later the cells have separated again (09:30). ( Video 2).

To better investigate how double-positive cells arose we performed time-lapse microscopy using wild-type trypanosomes expressing cytoplasmic DsRED or GFP, together with a GFP-tagged version of Histone 2B to visualize the nucleus. In order to track cells for extended periods we cultured them for 4 hours in medium without FBS, which causes them to adhere to the surface of the culture flask (see Materials and methods). These experiments revealed that the trypanosomes exchanged cytoplasmic proteins in both directions. The interacting cells became double positive within 10 minutes (the interval between images) and they could separate again ( Figure 2, Video 1 and Video 2). Some cells stayed connected for more than 7 hours ( Video 1), while others separated within 20 minutes ( Video 2). Neither cell-cell fusion nor nuclear exchange was observed, arguing against this phenomenon being mating.

**Figure 3.**
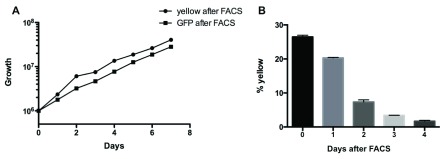
Double-positive cells lose one fluorescent protein over time. **A**: Growth curve of Δproc yellow cells after FACS and Δproc GFP cells after FACS. **B**: Double-positive Δproc cells were enriched by FACS and the percentage of yellow cells was measured by flow cytometry at daily intervals.

If proteins, but not DNA are exchanged between two cells, it would be expected that they lose one fluorescent protein once they separate. To test this we enriched for double-positive cells and monitored how long they retained both colours. After one round of FACS 26% of trypanosomes in the culture were double-positive ( Figure 3). Cells expressing GFP only were sorted as a control. Cell numbers and the percentage of yellow cells were determined every day. While the cell number more then doubled within the first 24 hours, the percentage of yellow cells decreased only slightly, indicating that the yellow cells still proliferated and were not simply overgrown by single positive cells ( Figure 3). After 3 days, however, the percentage of yellow trypanosomes returned to background level, consistent with turnover of the transferred proteins.

### Exchange of differentially localised proteins indicates involvement of the flagellum

**Figure 4.**
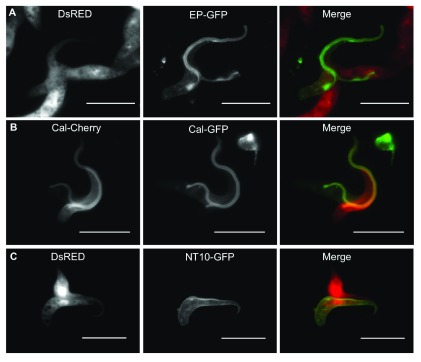
Exchange of different fluorescently tagged proteins indicates involvement of the flagellum. **A**: Interacting pair of trypanosomes expressing either DsRED (Δproc) or the GPI anchored surface protein EP-GFP (WT). **B**: Interacting pair of trypanosomes expressing the flagellar protein calflagin44-GFP (WT) or calflagin44-Cherry (WT). **C**: Interacting pair of trypanosomes expressing either cytoplasmic DsRED (Δproc) or the nucleoside transporter NT10-GFP (WT), which localises to the surface of the cell body, but not to the flagellum. The scale bar indicates 10μm.

The experiments described above show that procyclic form trypanosomes are capable of exchanging soluble proteins that are mainly present in the cytoplasm. To test whether surface proteins could also be exchanged we first used cells tagged with a GFP-procyclin fusion protein. In trypanosomes, newly synthesised GPI-anchored proteins gain access to the cell surface via the flagellar pocket. This is an invagination of the plasma membrane where the flagellum emerges from the cell body, and is the only known site of endo- and exocytosis. On exiting the pocket GPI-anchored proteins are distributed along the flagellum to the cell surface ^42^. In cells expressing GFP-procyclin the flagellar pocket is seen as an intensely fluorescent signal ( Figure 4A). When trypanosomes expressing cytoplasmic DsRED and GFP-procyclin were mixed together, we observed that DsRED was equally distributed between two interacting cells, but GFP-procyclin was only transferred to the flagellum of the recipient and not the rest of the cell surface ( Figure 4A). These results indicate that proteins on the outer leaflet of the plasma membrane can be transferred between cells, but transfer appears to be restricted to the flagellum.

Calflagin-44 is an acylated protein that is anchored to the flagellar membrane ^43^. To obtain more information on the involvement of the flagellum we generated stable transformants expressing calflagin-GFP and calflagin-mCherry fusion proteins. These were correctly localised to the flagellum, but the protein was sometimes also detected in the cell body, possibly due to its overexpression. When trypanosomes expressing calflagin-GFP and calflagin–mCherry were co-cultured, interacting pairs had both proteins in their flagella, but calflagins in the cell bodies were not exchanged ( Figure 4B). Based on these results we hypothesised that surface proteins that were excluded from the flagellum would not be exchanged. To test this we mixed cells expressing DsRED with trypanosomes expressing a GFP-tagged version of the nucleoside transporter NT10, which is restricted to the cell body ^44^. Interacting pairs from this mixture had DsRED distributed equally between the two cells while NT10-GFP remained only on one cell ( Figure 4C and Figure S2). Taken together, these data suggest that the flagellum is the primary site of interaction and protein exchange. In this context it is important to note that soluble GFP and DsRED are able to cross the diffusion barrier of the flagellar transition zone and are therefore present in the flagellar matrix as well as in the cytoplasm.

**Figure 5.**
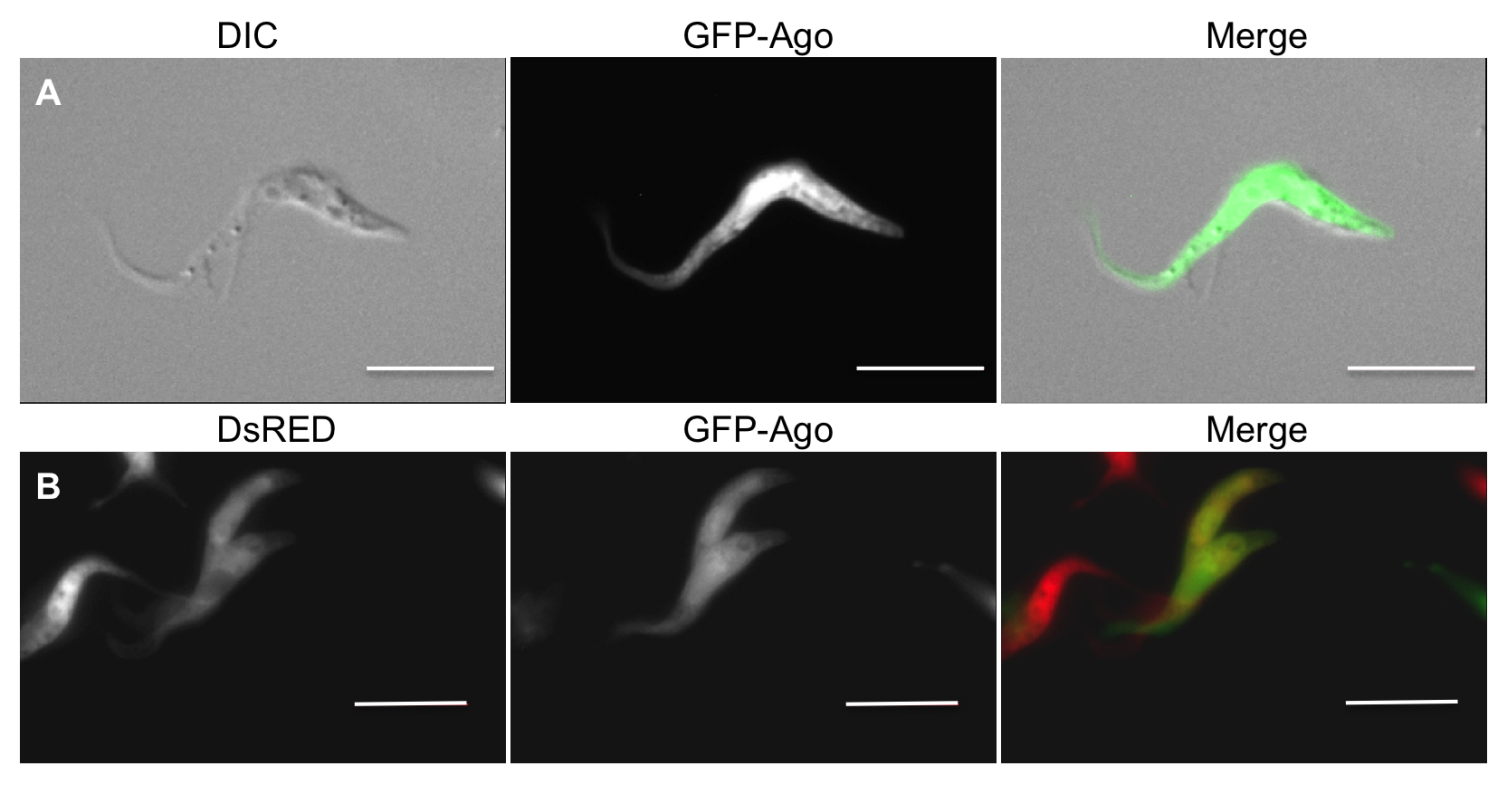
GFP-tagged Ago1 partially localises to the flagellum and can be exchanged between cells. **A**: Fluorescence microscopy of wild-type cells expressing GFP-Ago. **B**: Fluorescence microscopy of co-cultured wild-type cells expressing DsRED or GFP-Ago. Scale bars indicate 10μm.

RNA-binding proteins play a major role in regulating gene expression in trypanosomes. They can alter the stability or translational efficiency of individual mRNAs or mRNA cohorts ^45^, or they can silence them by RNA interference ^46^. To test if RNA-binding proteins could be exchanged between cells we used a tagged form of Argonaute (Ago) with GFP fused to its N-terminus (GFP-Ago). Cytoplasmic proteins >75kDa are normally excluded from flagella and cilia unless they contain targeting signals ^47– 49^. One rationale for choosing the GFP-Ago fusion was that the protein is 130 kDa and should therefore require active transport; the second rationale was that it is a catalytic component of the RNA-induced silencing complex. When cells expressed GFP-Ago, the protein was detected in the flagellum as well as in the cytoplasm ( Figure 5A). Moreover, when these cells were mixed with cells expressing DsRED, bidirectional exchange of both proteins was observed ( Figure 5B). In summary, trypanosomes have the capacity to transport Ago into the flagellum and to transfer it to another cell. This transfer could potentially reprogram gene expression in the recipient cell by transferring small interfering RNAs.

### Protein exchange can occur in less than one minute and fused cells are motile and continue to divide

**Figure 6.**
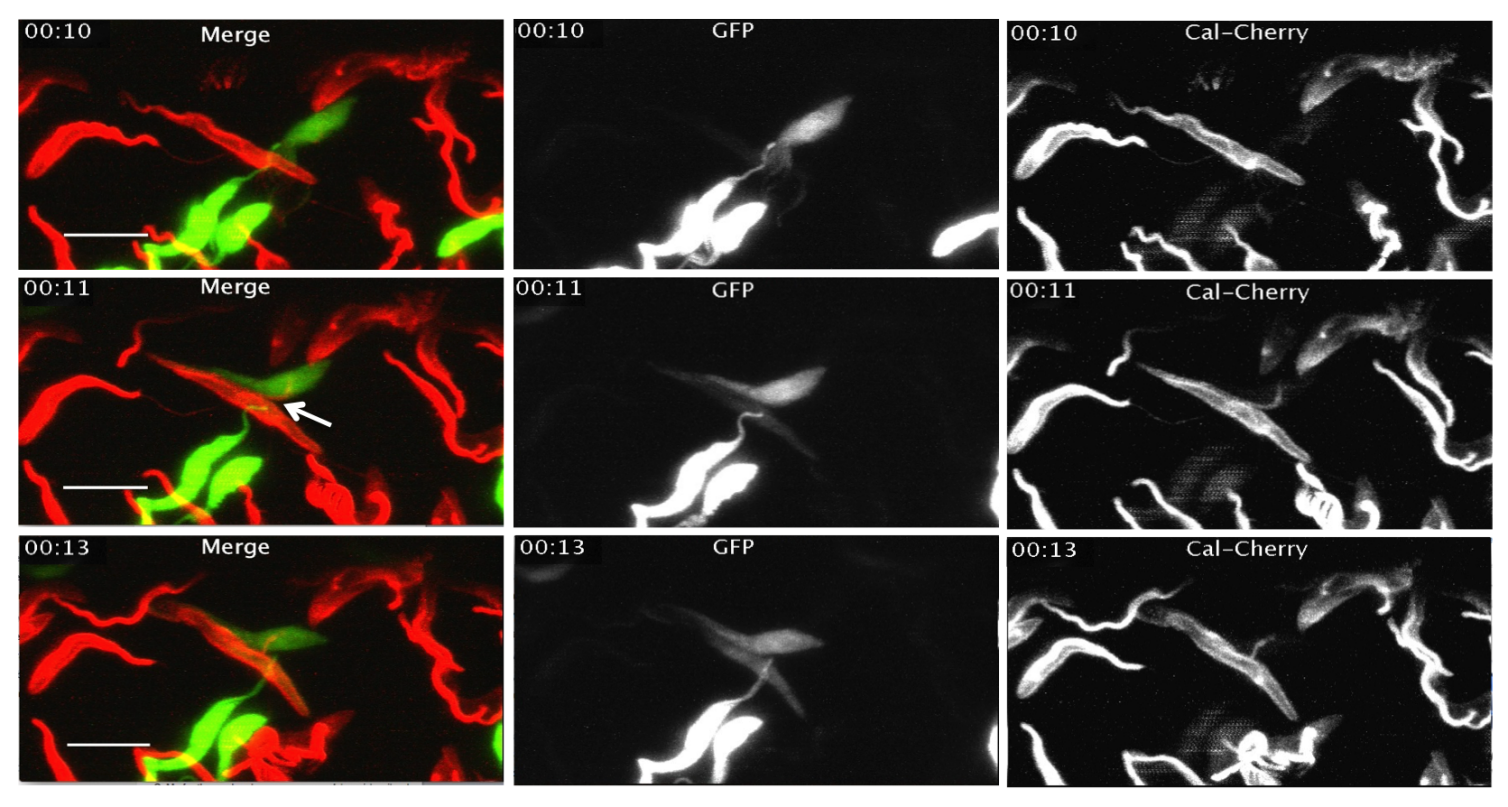
Time-lapse microscopy shows a rapid exchange of cytoplasmic and flagellar proteins. Still images from lattice light sheet fluorescence microscopy time-lapse video ( Video 3) of trypanosomes expressing either GFP (Δproc) or calflagin44-mCherry (WT). The time interval is indicated at the left upper corner of the image. The scale bar is 10μm. First image (00:10): cells are only positive for one fluorescent protein. Second image one minute later (00:11), calflagin44-mCherry is present on both cells, but GFP is detected only weakly in the second cell. Third image: two minutes later (00:13), GFP is equally distributed in both interacting cells.

To investigate protein exchange with higher temporal resolution, we performed time-lapse imaging with a lattice light sheet microscope ^50^ using an image acquisition interval of one minute. Again we mixed trypanosomes expressing cytoplasmic GFP or calflagin-mCherry. The exchange of calflagin-mCherry occurred in less than a minute ( Figure 6), while it took approximately 2–3 minutes until GFP was equally distributed between two cells ( Figure 6 and Video 3). These experiments further demonstrated that interacting pairs were still moving, indicating that the motility function of the flagellum was not impaired ( Video 3). Furthermore, complete divisions of interacting cells could be observed ( Video 4). While some interacting trypanosomes stayed together for an extended period of time, other interactions were very transient. The shortest interaction we observed was for 1–2 minutes ( Video 5).

### Fusion of flagellar membranes in double-positive cells

**Figure 7.**
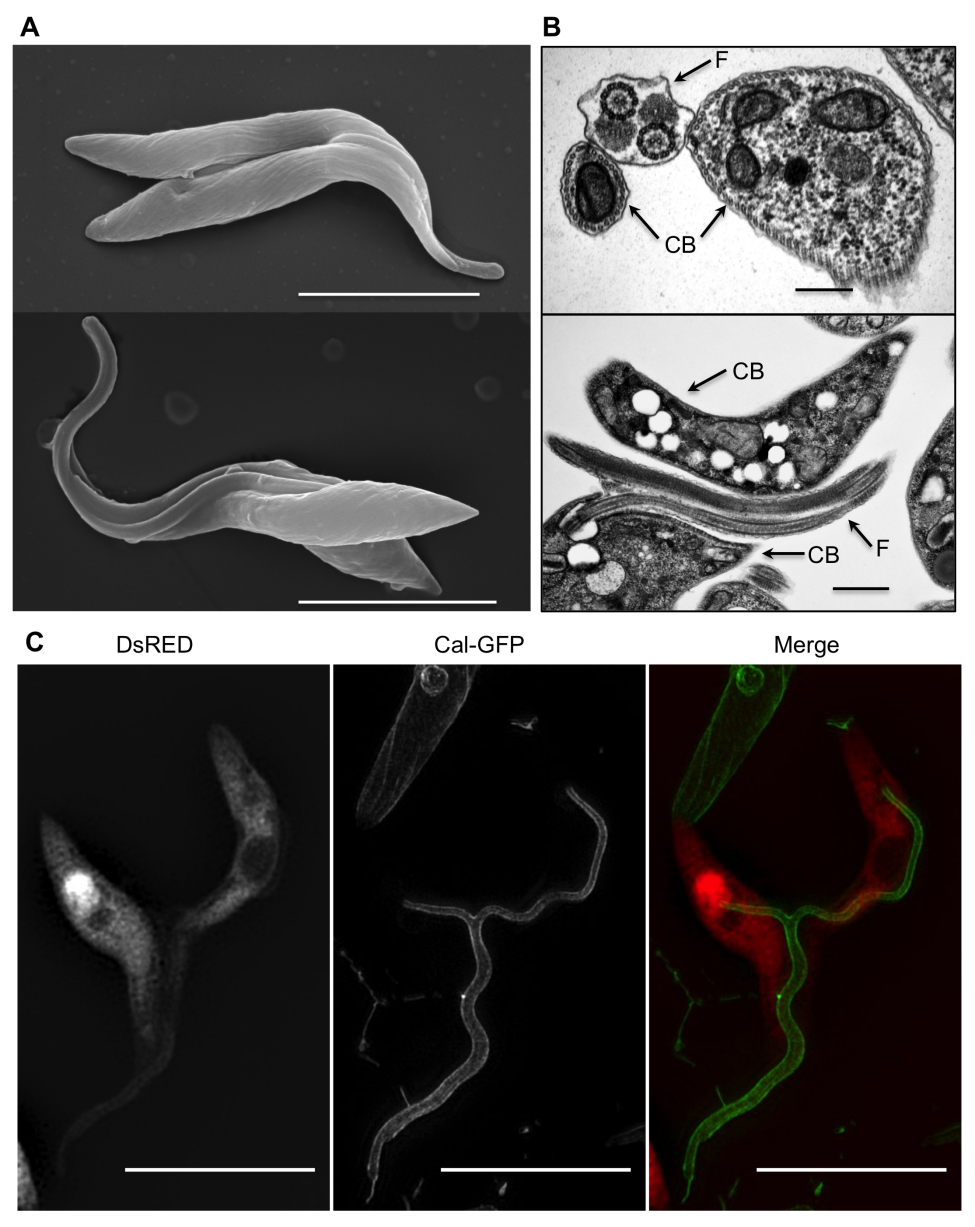
High-resolution images of interacting trypanosomes. **A**: Scanning electron microscopy of interacting trypanosomes (Δproc). The scale bar indicates 10μm. **B**: Transmission electron microscopy of fused flagella (Δproc). The scale bar indicates 0.5μm for the upper image and 1.5μm for the lower image; arrows indicate the cell-body (CB) and the flagellum (F). **C**: Structured illumination microscopy (SIM) of interacting trypanosomes with fused flagella. Trypanosomes were tagged with either DsRED (Δproc) or calflagin44-GFP (WT). The scale bar indicates 10μm.

The fact that trypanosomes exchange proteins anchored to the outer surface of the flagellar membrane (EP procyclin) or the inner surface (calflagin), suggested either fusion of these membranes or a short-range exchange of vesicles had taken place. To distinguish between these possibilities we performed electron microscopy (EM). Since only a few percent of a mixed population is double-positive, and only some of the cells interact at a given time-point, we again enriched for yellow cells by FACS. Using scanning EM we observed the same characteristic pairs of trypanosomes seen by fluorescence microscopy ( Figure 7A). The flagella of these pairs appeared thicker than the flagella of single cells, which could be compatible with fusion along their entire length. From these images alone, however, we could not conclude unambiguously that the membranes were fused; it remained possible that they were merely in very close contact. To better characterise the interaction between flagellar membranes we performed transmission EM ( Figure 7B and Figure S3). These images demonstrated unequivocally that pairs of cells shared a flagellum with two axonemes and two paraflagellar rods bounded by a single membrane ( Figure 7B and Figure S3A). This differs from what happens during cell division, in which the trypanosomes produce a separate new flagellum posterior to the old one. Thus, the most plausible explanation is that the two flagella have fused. We also documented examples of triple and quadruple fusions ( Figures S3B & S3C), albeit at a much lower frequency.

Structured Illumination Microscopy (SIM) provides a resolution doubling when compared to diffraction limited imaging and complements scanning EM data by delivering information on protein exchange. We therefore applied this method to co-cultures of trypanosomes expressing cytoplasmic DsRED or calfalgin-GFP. This confirmed that large parts of the flagella fuse into a single structure in double-positive cells ( Figure 7C). Once again, fusion of multiple cells could be observed ( Figure S4A). Pairs with a second flagellum forming on one cell, double-positive cells undergoing cytokinesis and fused cells giving rise to daughter cells were also seen ( Figures S4B & C). Taken together, these data suggest that flagellar fusion is a guided process that is part of normal cell functions.

Time-lapse imaging of wild-type trypanosomes expressing histone 2B-GFP together with cytosolic GFP or DsRED Images were acquired using a Nikon TE2000E-PFS microscope with a 60x objective. DIC and fluorescent images were taken every 10 minutes for 24 hours. Click here for additional data file. Copyright: © 2016 Imhof S et al. 2016 Data associated with the article are available under the terms of the Creative Commons Zero "No rights reserved" data waiver (CC0 1.0 Public domain dedication).

Time-lapse imaging of wild-type trypanosomes expressing histone 2B-GFP together with cytosolic GFP or DsRED Images were acquired using a Nikon TE2000E-PFS microscope with a 60x objective. DIC and fluorescent images were taken every 10 minutes for 24 hours. Click here for additional data file. Copyright: © 2016 Imhof S et al. 2016 Data associated with the article are available under the terms of the Creative Commons Zero "No rights reserved" data waiver (CC0 1.0 Public domain dedication).

Time-lapse imaging of trypanosomes expressing cytoplasmic GFP (Δproc) or calflagin-mCherry (WT) Performed using a lattice light sheet microscope with an image acquisition interval of one minute Click here for additional data file. Copyright: © 2016 Imhof S et al. 2016 Data associated with the article are available under the terms of the Creative Commons Zero "No rights reserved" data waiver (CC0 1.0 Public domain dedication).

Time-lapse imaging of trypanosomes expressing cytoplasmic GFP (Δproc) or calflagin-mCherry (WT) Performed using a lattice light sheet microscope with an image acquisition interval of one minute Click here for additional data file. Copyright: © 2016 Imhof S et al. 2016 Data associated with the article are available under the terms of the Creative Commons Zero "No rights reserved" data waiver (CC0 1.0 Public domain dedication).

Time-lapse imaging of trypanosomes expressing cytoplasmic GFP (Δproc) or calflagin-mCherry (WT) Performed using a lattice light sheet microscope with an image acquisition interval of one minute Click here for additional data file. Copyright: © 2016 Imhof S et al. 2016 Data associated with the article are available under the terms of the Creative Commons Zero "No rights reserved" data waiver (CC0 1.0 Public domain dedication).

## Discussion

Our results demonstrate that procyclic culture form trypanosomes can use their flagella as conduits for exchanging proteins with other individuals in a population. Cells that become double-positive for GFP and DsRED revert to being single-positive over a period of 72 hours, in agreement with the observation that no DNA is transferred and that these cells are not genetic hybrids. Protein exchange entails fusion of the flagellar membrane, which can be partial or along the entire length. In common with the fusion of plasma membranes between myoblasts ^51^ or of synaptic vesicles with the plasma membrane of neurons in multicellular organisms ^52^, flagellar fusion in trypanosomes is calcium-dependent.

Protein exchange between trypanosomes is bidirectional and can occur in under a minute. This limit of temporal resolution in our experiments was dictated by the need to capture many fields of view in order to observe rare fusion events in a population across time. There are several indications that flagellar fusion does not impair cell function and that it can be reversed. Live imaging revealed that paired cells are motile; pairs can remain together for hours, and even give rise to progeny while in this state, or they can separate within a minute. Thus, the double-positive cells observed 24 hours after mixing could be the result of a transient fusion or they could be daughter cells of previously fused trypanosomes.

Wild-type cells that are double-positive usually occur at extremely low frequencies in co-cultures (≈1%), implying that only a small proportion of the population is fusion competent at any one time. These numbers might be an under-estimate, however, if interactions are more fleeting and less protein is transferred between cells. Furthermore, protein exchange between two green cells or two red cells would not be detected. The percentage of double-positive cells increased when cells were washed and supplied with fresh serum, suggesting that factors that inhibit fusion accumulate in the medium. It is not clear whether both cells need to be in a fusion-competent state or if one cell suffices. In the course of live imaging we noted that paired cells seemed to attract additional cells to fuse ( Video 1) and examples of 3 or more fused flagella were observed by transmission EM and SIM ( Figure S3 and Figure S4). Many proteins, including several potential signal transducers, are localized to different flagellar domains ^22, 53^. The Δproc mutant fused more readily than the wild type, which might reflect increased accessibility of components on the flagellar surface. Extracellular vesicles produced by bloodstream form trypanosomes have a sparser variant surface glycoprotein coat than the cell surface, and it was hypothesised that this might influence their fusogenic properties ^24^.

The trypanosome flagellum has a different lipid and protein composition than the cell body, providing a certain selectivity of exchanged proteins ^21, 53, 54^. Both soluble and membrane-associated proteins can be translocated between cells provided that they gain access to the flagellum. In contrast, a polytopic membrane protein that is restricted to the surface of the cell body is not transferred between trypanosomes. Although we do not know why trypanosomes exchange cell contents, several possibilities spring to mind. Direct transfer would prevent proteins being diluted or destroyed in the extracellular milieu and might also prevent activation of an antimicrobial response by the host. Sampling each other's proteins and metabolites might enable cells within a population to synchronise. Alternatively, healthy cells might rescue damaged or stressed cells by providing missing components. In this context, two recent publications have shown that tight cell-cell interactions ^55^ or nanotubes ^56^ can enable two bacterial species to exchange missing nutrients. Although double-positive cells seem to proliferate normally after protein exchange, implying that the interaction is relatively benign, we cannot exclude that this mechanism is used by one trypanosome to exploit resources of the other or even to deliver harmful cargo to a competing cell. A further possibility is that exchange might deliver signals for differentiation. Even if protein exchange is transient, it might be sufficient to reprogram the recipient cell. For example, it was shown that inducing expression of a single RNA-binding protein, RBP6, in procyclic (midgut) forms is enough to drive their differentiation to the life-cycle stages normally found in the salivary glands ^57^.

The flagellar proteome of procyclic forms of *T. brucei* contains a variety of metabolic enzymes, peptidases, heat shock proteins and RNA-binding proteins, all of which have the potential to be exchanged upon fusion ^53^. It has not been established whether mRNA is present in the flagellum, but poly(A)-binding protein 1 has been detected in the flagellar proteome ^53^. Furthermore, when Ago is exchanged between cells, small interfering RNAs (siRNAs) might be carried over as cargo. At present, however, siRNAs cannot be visualised directly by *in situ* hybridisation, because of issues with sensitivity and specificity.

To date, we have not detected double-positive procyclic (midgut) forms in tsetse flies. GFP and its derivatives appear to be mildly toxic for procyclic forms *in vivo* and trypanosomes expressing it are often overgrown during co-infections ^40^. This could potentially bias the outcome. It is also possible that we observe an extreme form of flagellar fusion in culture and that many interactions between procyclic forms might be too brief and the amount of exchanged protein too low to be detected unequivocally *in vivo*. Double-positive trypanosomes definitely occur in the salivary glands of tsetse, and have been construed as evidence of mating ^35, 36, 41^. The expression of meiotic markers by trypanosomes in the glands ^37^ makes it highly likely that this is indeed the site where genetic hybrids form. Nevertheless, several factors make it challenging to distinguish between mating and protein exchange. Since salivary gland forms of *T. brucei* cannot be cultured, it is impossible to follow double-positive cells over a longer period of time to monitor loss or retention of fluorescent proteins. The low frequency of mixed salivary gland infections and low abundance of yellow cells also make these studies extremely labour intensive. Several previous observations could indicate that a proportion of double-positive cells in the fly are not genetic hybrids. The current model of genetic exchange in trypanosomes involves meiosis and the formation of haploid gametes, which subsequently fuse together ^38^. When flies were co-infected with cells coding for the meiotic marker HOP1 fused to YFP and cells expressing mRFP, trypanosomes positive for both proteins could be detected, indicating that protein exchange occurred before the formation and fusion of gametes (as also mentioned by the authors of the study; ^37^). Double-positive *Leishmania major* cells have also been detected during co-infections of sand flies, which again has been taken as evidence of genetic exchange ^58^. It was not possible to isolate their progeny, however, and an alternative explanation might be that these cells had only exchanged proteins, but not DNA.

The list of functions attributed to flagella, in addition to motility, is constantly expanding. Nanotubular structures budding from the flagellar membrane of bloodstream form trypanosomes give rise to extracellular vesicles that are able to transfer proteins between cells ^24^. Protein transfer by extracellular vesicles might reflect a broader form of communication within the trypanosome population, while the protein exchange by flagellar fusion described here could be used for direct, contact-dependent communication between two cells. In addition ectosomes released from the flagella of *Chlamydomonas rheinhardtii* enable daughter cells to hatch from their mother cell ^59^. The flagella of *T. brucei, Leishmania* spp, and *Chlamydomonas* are related to the cilia of higher eukaryotic cells and many proteins involved in intraflagellar transport (IFT) are conserved ^60^. IFT seems to have additional functions that extend to cells without cilia; it has been linked to exocytosis ^61^, and IFT proteins have been localised to the synapse between T cells and antigen-presenting cells ^62^. Very recently a new type of microtubule-based nanotube was described ^63^; this is dependent on IFT proteins for its function in the *Drosophila* germline. It was proposed that this structure provides selectivity for receptor-ligand interactions, but it was not reported whether membrane fusion occurred or if proteins were transferred from one cell to the other. In this context it is worth noting that attachment of trypanosomes in the salivary glands involves remodeling of both the host epithelial membranes and the parasite flagellar membrane, indicating that the flagellum may be capable of transmitting and receiving signals to and from the host cells. We consider it worth exploring if other flagellated parasites, as well as the sensory cilia of higher eukaryotes also possess the ability to mediate protein exchange between cells.

## Materials & methods

### Reagents

Unless otherwise specified, chemicals were obtained from Sigma-Aldrich, Switzerland. Oligonucleotide primers were synthesised by Microsynth AG (Balgach, Switzerland). Enzymes were purchased from New England Biolabs.

### Trypanosomes

Procyclic forms of *T. b. brucei* AnTat 1.1 ^64^ and genetically manipulated derivatives were used in this study. The deletion mutant Δproc ^40^ was described previously. Trypanosomes were cultured at 27°C in SDM-79 (Amimed, Cat. No. 9-04V01M) supplemented with 10% heat inactivated foetal bovine serum ^65^.

Conditions for double-positive cells: 5 × 10 ^6^ cells expressing DsRED were mixed with 5 × 10 ^6^ cells expressing GFP and centrifuged at 1300g for 6 minutes in a 15ml Falcon tube. The cells were resuspended in 10ml phosphate-buffered saline and centrifuged again. The washed trypanosomes were resuspended in 2.5ml SDM-79 with 10% fresh FBS and cultured overnight at 27°C in one well of a six-well plate. The cells tend to adhere weakly to the bottom of the well, so before analysis they were gently flushed loose with a 1ml pipette.

### Constructs and generation of stable transformants

Stable transformation of procyclic form trypanosomes was performed as described ^66^. To clone the plasmids pG-EGFP-Blast-ΔLII and pG-DsRED-Blast-ΔLII, pG-EGFP-ΔLII ^67^ and pG-DsRED-ΔLII ^68^ were digested with the restriction enzymes NheI and ClaI to cut out the neomycin resistance gene. The blasticidin resistance gene was amplified by PCR using the primers NheIBlast (GC TAGCTAGCATGGCCAAGCCT) and ClaBlast (CC ATCGATACTCACAGCGACTA) and pC-EP2-ΔLII-Blast as template ^40^ the product was digested with NheI and ClaI and ligated into pG-EGFP-ΔLII and pG-DsRED-ΔLII.

The mCherry coding region was amplified by PCR using the plasmid CWP1:mCherry (a gift from Adrian Hehl, Zürich University) as a template and the primers mCherryfor (TT ACCGGTCATGGTGAGCAAGGGCG) and mCherryrev (TT GGATCCCGGGCTTGTACAGCTCGTCCATG). The PCR product was digested with BamHI and AgeI and ligated into BamHI/AgeI digested pG-EGFP-ΔLII, replacing EGFP by mCherry. To change the selectable marker of pG-mCherry-ΔLII from neomycin-resistance to phleomycin-resistance the plasmid G-BIL4-phleo was digested with XbaI and NotI to excise the phleomycin resistance gene. The phleomycin resistance gene was then ligated into the XbaI/NotI-digested pG-mCherry-ΔLII.

To tag Calflagin44 at its C-terminus with EGFP or mCherry, the coding region of Cal44 was amplified by PCR using the primers Cal44for (AT AAGCTTATGGGTTGCTCTGCATCG) and Cal44rev (AT GTCGACGATTACCTTCATTTGCTCC) and genomic DNA as the template. The PCR product was cloned into the pGEM-T Easy Vector System (Promega), subsequently excised with HindIII and SalI, then ligated into HindIII/SalI digested pG-EGFP-Blast-ΔLII and pG-mCherry-Phleo-ΔLII. To tag Ago1 at its N-terminus the Ago1 coding region was amplified by PCR using the primers Ago1SmaFor ( CCCGGGATGTCTGACTGGGAAC) and Ago1SmaRev ( CCCGGGTTATAGATAATGCATTGTTG) and the product was cloned into the pGEM-T Easy Vector System (Promega). The plasmids pGEM-Ago1 and pG-EGFP-ΔLIIγ ^69^ were digested with the restriction enzyme SmaI and the coding region of Ago1 was ligated into pG-EGFP-ΔLIIγ, correct integration was confirmed by sequencing.

The constructs pG-H2B-GFP-ΔLII and pG-NT10-GFP-ΔLII were described previously ^44, 69^.

### Fluorescence microscopy

Cells were washed in phosphate buffered saline (PBS), spread on a microscope slide and mounted with Moviol. Images were taken with a Leica DFC360FX monochrome CCD (charge-coupled-device) camera mounted on a Leica DM5500 B microscope with a 100x oil immersion objective and analysed using LAS AF 1.0 software (Leica).

Structured illumination microscopy (SIM) was performed as described in 70. Cells were fixed for 20 minutes at room temperature with 4% paraformaldehyde and 0.5% glutaraldehyde. For each axial plane of a 3D stack, raw SIM images were acquired at five phase steps spaced by 2π/5 of the illumination pattern period, and this process was repeated with the excitation pattern rotated by ±120° with respect to the first orientation. The axial stepping size was set to 160 nm. The raw data was reconstructed into the 3D super-resolution image based on the algorithm described in 71. In our study, when the result was Fourier transformed back to real space, we applied a gamma apodization function *A*( *k*)=1–( *k*/ *k*_max_) ^*γ*^, with *γ* = 0.4, rather than the traditional triangle apodization *A*( *k*)=1– *k*/ *k*_max_^71^, where *k _max_* is the maximum support of the expanded optical transfer function (OTF). Therefore, the higher spatial frequencies were not suppressed more than necessary. Furthermore, we strictly follow the azimuthally dependent maximum support *k _max_(θ)* to define the endpoint of the apodization function. All of these provided a better suppression of the ringing artifacts associated with Fourier transformation. After reconstruction, SIM images achieve 110 nm lateral, 350 nm axial resolution.

### FACS and flow cytometry

Trypanosomes (Δproc) expressing either DsRED or GFP were washed in PBS and co-cultured in SDM-79 with 10% fresh FBS for 8 hours at a density of 10 ^7^ cells/ml. Then the cells were transferred into PBS containing 2% FBS at a density of 1.5×10 ^7^ cells/ml for sorting. Sorting was performed with a BD FACS ARIA III (BD Biosciences) equipped with a 130μm nozzle running with 10 psi pressure, flow liquid was PBS. The software used was BD FACS DIVA 6.0 (BD Biosciences). To detect GFP a 488nm blue laser with a 530/30nm bandpass filter was used, to detect DsRED a 561 yellow-green laser with a 610/20 bandpass filter was used. Double-positive cells were sorted into SDM-79 10% FBS.

Flow cytometry was performed with living cells in PBS, 10 ^4^ cells were analysed using a FACSCalibur (BD Biosciences) and analysed with CellQuest Pro (version 5.1.1).

### Electron microscopy

***Transmission electron microscopy.*** Trypanosomes were washed briefly in PBS, then fixed in 2.5% glutaraldehyde in 100 mM sodium cacodylate buffer (pH7.3) for 2 hours at room temperature, followed by post-fixation in 2% OsO4 in cacodylate buffer, pH 7.3. After three washes in distilled water, samples were pre-stained in saturated uranyl acetate solution in distilled water for 30 min at room temperature, extensively washed in distilled water, and dehydrated by stepwise incubation in ethanol (30%-50%-70%-90%-100%). Parasites were then embedded in EPON812 resin as described previously ^72^. Following polymerisation of the resin at 60°C for 24 h, sections of 80 mm thickness were cut on a ultramicrotome (Reichert & Jung, Vienna, Austria), placed onto 300 mesh formvar-carbon-coated nickel grids (Plano, Wetzlar, Germany), and sections were stained with uranyl acetate and lead citrate ^40^. Specimens were viewed on a Phillips 400 TEM operating at 60 kV.

***Scanning electron microscopy.*** Trypanosomes were washed briefly in PBS, then fixed in 2.5% glutaraldehyde in 100 mM sodium cacodylate buffer, pH 7.3, for 2 hours at room temperature, followed by post-fixation in 2% OsO4 in cacodylate buffer, pH 7.3. They were then extensively washed in water, dehydrated by stepwise incubation in ethanol (30%-50%-70%-90%-100%), and two short washes in 50µl hexamethyl-disilazane. Trypanosomes were then taken up in a small volume of hexamethyl-disilazane, and were allowed to settle down on glass coverslips and were air-dried under a fume hood. They were then sputter-coated with gold ^73^ and inspected on a JEOL 840 scanning electron microscope operating at 25 kV.

### Time-lapse imaging

To immobilise the trypanosomes approximately 3×10 ^6^ cells of each clone were mixed and centrifuged for 6 minutes at 1300g. The cell pellet was washed with 10 ml PBS and centrifuged again using the same parameters as above. The cells were resuspended in 1 ml SDM-79 without FBS and incubated for 4 hours at 27°C in a glass bottom culture dish (Willco Wells, the Netherlands). During this time the trypanosomes adhered to the bottom of the dish and were immobilised. After 4h 100μl FBS was added to the cells and the time-lapse imaging was started immediately using a Nikon TE2000E-PFS microscope with a 60x objective. DIC and fluorescent images were taken every 10 minutes for 24 hours.

The 4D lattice light sheet measurements were performed using a microscope described previously ^50^. This system illuminates the sample with a massively parallel array of coherently interfering beams comprising a non-diffracting 2D optical lattice. This creates a coherent, spatially structured light sheet that is then dithered to create uniform excitation in a ~600 nm thick plane across the entire field of view. In order to capture the statistically rare fusion events in trypanosome samples, 3D images were acquired from 15 fields of view every 60 seconds for a total duration of 9h12min. Raw data were deconvolved via a 3D iterative Lucy-Richardson algorithm in Matlab version 2013b (The Mathworks, Natick, MA) utilizing an experimentally measured point spread function using 100 nm fluorescent beads (Fluospheres, ThermoFisher Cat #: F8803). Movies were generated using ImageJ (Version 1.49m).

Raw data for Figure 1 Click here for additional data file. Copyright: © 2016 Imhof S et al. 2016 Data associated with the article are available under the terms of the Creative Commons Zero "No rights reserved" data waiver (CC0 1.0 Public domain dedication).

Raw data for Figure 4 Click here for additional data file. Copyright: © 2016 Imhof S et al. 2016 Data associated with the article are available under the terms of the Creative Commons Zero "No rights reserved" data waiver (CC0 1.0 Public domain dedication).

Raw data for Figure 5 Click here for additional data file. Copyright: © 2016 Imhof S et al. 2016 Data associated with the article are available under the terms of the Creative Commons Zero "No rights reserved" data waiver (CC0 1.0 Public domain dedication).

Raw data for Figure 7C Click here for additional data file. Copyright: © 2016 Imhof S et al. 2016 Data associated with the article are available under the terms of the Creative Commons Zero "No rights reserved" data waiver (CC0 1.0 Public domain dedication).

Raw data for Figure S2 Click here for additional data file. Copyright: © 2016 Imhof S et al. 2016 Data associated with the article are available under the terms of the Creative Commons Zero "No rights reserved" data waiver (CC0 1.0 Public domain dedication).

Raw data for Figure S4 Click here for additional data file. Copyright: © 2016 Imhof S et al. 2016 Data associated with the article are available under the terms of the Creative Commons Zero "No rights reserved" data waiver (CC0 1.0 Public domain dedication).

Raw data for Video 1 Click here for additional data file. Copyright: © 2016 Imhof S et al. 2016 Data associated with the article are available under the terms of the Creative Commons Zero "No rights reserved" data waiver (CC0 1.0 Public domain dedication).

Raw data for Video 2 Click here for additional data file. Copyright: © 2016 Imhof S et al. 2016 Data associated with the article are available under the terms of the Creative Commons Zero "No rights reserved" data waiver (CC0 1.0 Public domain dedication).

## Data availability

The data referenced by this article are under copyright with the following copyright statement: Copyright: © 2016 Imhof S et al.

Data associated with the article are available under the terms of the Creative Commons Zero "No rights reserved" data waiver (CC0 1.0 Public domain dedication). 

*F1000Research*: Dataset 1. Raw data for Figure 1, 10.5256/f1000research.8249.s120067 ^74^

*F1000Research*: Dataset 2. Raw data for Figure 4, 10.5256/f1000research.8249.s120068 ^75^

*F1000Research*: Dataset 3. Raw data for Figure 5, 10.5256/f1000research.8249.s120069 ^76^

*F1000Research*: Dataset 4. Raw data for Figure 7C, 10.5256/f1000research.8249.s120070 ^77^

*F1000Research*: Dataset 5. Raw data for Figure S2, 10.5256/f1000research.8249.s120071 ^78^

*F1000Research*: Dataset 6. Raw data for Figure S4, 10.5256/f1000research.8249.s120072 ^79^

*F1000Research*: Dataset 7. Raw data for Video 1, 10.5256/f1000research.8249.s120075 ^80^

*F1000Research*: Dataset 8. Raw data for Video 2, 10.5256/f1000research.8249.s120077 ^81^

Access to additional time-lapse imaging data may be arranged by contacting the corresponding author.

*Figshare*: Time-lapse imaging of wild-type trypanosomes expressing histone 2B-GFP together with cytosolic GFP or DsRED. doi: 10.6084/m9.figshare.3126292.v1 ^82^

*Figshare*: Time-lapse imaging of wild-type trypanosomes expressing histone 2B-GFP together with cytosolic GFP or DsRED. doi: 10.6084/m9.figshare.3126352.v1 ^83^

*Figshare*: Time-lapse imaging of trypanosomes expressing cytoplasmic GFP (Δproc) or calflagin-mCherry (WT). doi: 10.6084/m9.figshare.3126355.v1 ^84^

*Figshare*: Time-lapse imaging of trypanosomes expressing cytoplasmic GFP (Δproc) or calflagin-mCherry (WT). doi: 10.6084/m9.figshare.3126412.v1 ^85^

*Figshare*: Time-lapse imaging of trypanosomes expressing cytoplasmic GFP (Δproc) or calflagin-mCherry (WT). doi: 10.6084/m9.figshare.3126415.v1 ^86^
