# Supplementary material for: Flagellar membrane fusion and protein exchange in trypanosomes; a new form of cell-cell communication?
Source: F1000Res. 2016 Apr 14;5:682. [Version 1] doi: 10.12688/f1000research.8249.1 (PMC4870996; doi:10.12688/f1000research.8249.1)
Supplement: Supplementary file 14 [file f1000research-5-8871-s0000.tgz › c7baea27-e89b-4015-8f61-7e2053237bb7.docx]

**Figure S1:** Flow cytometry of Δproc cells expressing GFP or DsRED, co- cultured for 24 hours in the presence of 10% fresh FBS and different additives. All treatments were performed in triplicate, error bars indicate standard deviations. Cells immediately mixed before analysis were used to determine the background (0h mix).

**Figure S2:** Structured illumination microscopy of co-cultured cells expressing either DsRED (Δproc) or NT10-GFP (WT). Scale bar indicating 10μm.

**Figure S3:** Transmission electron microscopy of multiple fused trypanosomes (Δproc).

**Figure S4:** Structured illumination microscopy of co-cultured trypanosomes either expressing DsRED (Δproc) or Cal-GFP (WT). A: Four trypanosomes with fused flagella. Scale bars indicate 10μm. B: Double-positive pair, a second flagellum is present on one cell, indicating that it is progressing through the cell-cycle. C: Two double-positive cells in late cytokinesis while one is fused to a third cell.
