# Supplementary material for: Flagellar membrane fusion and protein exchange in trypanosomes; a new form of cell-cell communication?
Source: F1000Res. 2016 Apr 14;5:682. [Version 1] doi: 10.12688/f1000research.8249.1 (PMC4870996; doi:10.12688/f1000research.8249.1)
Supplement: Raw data for Video 1 [file f1000research-5-8871-s0007.tgz › de60394d-3605-4e9c-bc69-3eac74a59713.zip!/Raw data Video 1/Legends for raw data.docx]

**LEGENDS FOR RAW DATA FOR VIDEOS 1 & 2**

Sequential frames are presented as jpg files.

C1: phase contrast; C2: GFP; C3: dsRED
