# Supplementary material for: Flagellar membrane fusion and protein exchange in trypanosomes; a new form of cell-cell communication?
Source: F1000Res. 2016 Apr 14;5:682. [Version 1] doi: 10.12688/f1000research.8249.1 (PMC4870996; doi:10.12688/f1000research.8249.1)
Supplement: Peer review history

# Peer review: Referee response for version 1

**DOI:** 10.5256/f1000research.8871.r13625

**Published:** 2016-04-28

**Authors:** Peter Satir

**Affiliations:** 1 Department of Anatomy and Structural Biology, Albert Einstein College of Medicine, Bronx, NY, USA

This article describes an interesting, transient interaction between flagella (cilia) of procyclic Trypanosomes, involving exchange of flagellar membrane and cytosolic molecules that can enter the flagellum, that can form quickly and last for some time, sometimes resulting in fusion of the two flagella. Experimental evidence for this phenomenon is shown by mixing populations of cells containing plasmids that produce different fluorescently labelled molecules (GFP and Ds RED) of interest. Originally a few yellow fluorescing cells were seen – indicative of interaction. The system was then refined for study by time lapse and electron microscopy. The data are well presented. The demonstration that molecules that cannot enter the flagellum are not transferred (Figure 4) is particularly nice. There is a suggestion that small RNAs could be transferred which might give the phenomenon added significance. 

The authors also suggest that this could be a new, more general, means of  cell communication involving cilia. It is different from usual ciliary pairing well studied during mating reactions, for example in Tetrahymena or Euplotes. Mating of course eventually involves intercellular bridges in protozoa, so the sentence in the introduction that states that no intercellular bridges are known in protozoa is not correct. In broadening their discussion to mammalian and other cilia, the authors might note the work of Ott *et al. *(2012) (see also Jackson’s gloss) on MDCK and other cells, which also describes cilia pairing, possibly for communication, but without fusion. It would be useful to know how the cells here separate after flagellar fusion.

The videos of the experiments are mostly useful in dynamically emphasizing the points made in the Figs, although some (i.e. Video 3) can be hard to follow. The previous reviewer commented on Video 2 where a cell ends up 4 nuclei – actually  nuclei of both interacting cells divide (one begins with two nuclei) although the cells themselves do not. Could this be important?

These few discussion points should add to the interest of this article.

I have read this submission. I believe that I have an appropriate level of expertise to confirm that it is of an acceptable scientific standard.

## References

[1] OttCEliaNJeongSYInsinnaCSenguptaPLippincott-SchwartzJ: Primary cilia utilize glycoprotein-dependent adhesion mechanisms to stabilize long-lasting cilia-cilia contacts. *Cilia*.2012;1(1) : 10.1186/2046-2530-1-3 3 10.1186/2046-2530-1-3 23351752PMC3541541

[2] JacksonP: Cilia develop long-lasting contacts, with other cilia. *Cilia*.2012;1(1) : 10.1186/2046-2530-1-5 10.1186/2046-2530-1-5 23351843PMC3541542

# Peer review: Referee response for version 1

**DOI:** 10.5256/f1000research.8871.r13386

**Published:** 2016-04-26

**Authors:** Scott M. Landfear

**Affiliations:** 1 Department of Molecular Microbiology and Immunology, Oregon Health and Sciences University, Portland, OR, USA

This article by Imhof *et al*. describes the unanticipated and intriguing observation that flagella of procyclic form African trypanosomes are capable of fusing and exchanging both membrane bound and cytosolic proteins between the interacting cells. Flagellar fusion occurs in ~1% of cells under normal culture conditions, but fusion can be increased up to ~5% of the population with appropriate manipulations. For proteins to be transferred from one cell to the next, they must have access to the flagella. A nucleoside transporter that is localized to the cell body but not flagellar membrane is not transferred, whereas proteins bound to the inner our outer face of the flagellar membrane, or non-membrane proteins, can be transferred. Striking transmission electron micrographs are shown in which two or more flagella are fused, and the corresponding axonemes and paraflagellar rods are encompassed within a single flagellar membrane. Fusion of trypanosomes expressing two different fluorescently labeled proteins can occur within 1 minute of mixing and can persist for minutes or for hours. Parasites fused by their flagella remain motile and are able to undergo cell division, producing two daughter cells that are both dually labeled. This phenomenon is clearly distinct from mating-associated fusion that has been observed in tsetse fly salivary gland stage trypanosomes, because the cell bodies and nuclei do not merge during flagellar fusion, but they do fuse during mating.

As the authors discuss, the major mystery is what purpose this behavior serves for parasites. While the phenomenon has been observed only in culture, it seems likely that the process will also occur in trypanosomes within tsetse flies. Furthermore, recent reports in both trypanosomes and other eukaryotes have underscored the role of the flagellum in transfer of proteins and membranes between cells. Recently, blood stream trypanosomes have been shown to secrete tubules and vesicles that can fuse with host red blood cells, and *Chlamydomonas reinhardtii* release from their flagellar tips vesicles that carry protein cargo. Thus flagella play a variety of biological roles, many involving the flagellar membranes, that were not anticipated even a few years ago. This paper raises the prospect that flagella and cilia in various organisms may be involved in directed exchange of proteins and should alert the scientific community to another possible broader function of these organelles.

Minor Points. In Video 2, one of the cells that has undergone exchange of the two fluorescently labeled proteins acquires 4 nuclei without undergoing division. Do the authors see other examples of this phenomenon, and do they have any comments about this behavior?

I have read this submission. I believe that I have an appropriate level of expertise to confirm that it is of an acceptable scientific standard.
